# Different Fc scaffolds enhance the breadth of *in vitro* neutralization of the same Fab against different Rotavirus strains

**DOI:** 10.3389/fimmu.2025.1709107

**Published:** 2026-01-09

**Authors:** Mabel Rocio Miranda-Echagüe, Giacomo Vezzani, Elena Morandi, Melania Della Peruta, Mirko Scordio, Teresa Anne Clarisse Reyes, Davide Oldrini, Miren Iturriza-Gómara, Rebecca Jo Loomis, Omar Rossi

**Affiliations:** 1 Glaxo Smith Kline (GSK), Vaccines Institute for Global Health (GVGH), Siena, Italy; 2 Fondazione Biotecnopolo di Siena, Siena, Italy

**Keywords:** immunology, mAbs scaffold, neutralization assay, rotavirus, vaccines

## Abstract

Rotaviruses are the primary cause of severe dehydrating diarrhea in infants and young children globally. Currently, several oral rotavirus vaccines are available; however, they have shown reduced effectiveness and quicker waning of protection in low- and middle-income countries (LMICs) compared to high-income countries (HICs). Both neutralizing and non-neutralizing antibodies against the middle (VP6) and outer layer capsid proteins (VP4 and VP7) are detected after infection, with higher titers being linked to disease protection. Historically, human derived rotavirus-specific monoclonal antibodies (mAbs) have been produced in an IgG1 scaffold, irrespective of whether their native scaffold was IgG or IgA. To explore the impact of antibody scaffolds on their functional activity we expressed mAbs targeting epitopes on VP8* or VP7 viral proteins in IgG1, IgG2, IgG3, IgG4, IgA1, and IgA2 scaffolds (the latter either in a monomeric or in a dimeric IgA form). The mAbs were characterized for their binding affinity to the viral target and their functionality was evaluated in an *in vitro* neutralization assay using three different rotavirus strains (G1P[8], G2P[4], and G9P[8]), representing homologous and heterologous specificities. Our findings revealed that mAbs targeting the same epitope exhibited varying neutralization activities when expressed in different scaffolds and suggested an enhanced breadth of neutralization activity in the context of an IgA scaffold. These data suggest that scaffolds impact the ability of antibodies to neutralize rotavirus. These findings can assist vaccine design informing the use of different technologies or adjuvants to elicit more effective antibody classes and subclasses.

## Introduction

Rotaviruses are the leading causes of diarrhea and mortality in children under five years old, worldwide (1). Although live-attenuated oral vaccines are available and are highly effective in high income countries, their effectiveness in low- and middle-income countries (LMICs) is suboptimal (50% to 70%) with waning of protection in the second year of life (2). Consequently, the majority of the 169,000 annual deaths due to Rotavirus infections are reported in LMICs (3).

Infection occurs through oral route, eliciting both secretory immunoglobulin A (sIgA) and systemic IgA and IgG responses, directed mainly against the intermediate layer protein VP6, and the neutralizing antibodies against the outer capsid proteins VP4 and VP7 (4). Upon infection, the VP4 spike protein undergoes cleavage into 2 different components, VP8* and VP5*. Antibodies targeting the VP8* head tend to be type-specific, while VP5* induces more cross-reactive neutralizing antibody responses (5). High systemic IgA and IgG titers have been correlated with protection from Rotavirus infection and vaccine efficacy (6, 7), however, no correlate of protection (CoP) has been definitively established.

Although the production of rotavirus-specific antibodies is crucial for inducing protection, it is not clear if the different Abs subclasses play different roles in protection; moreover, it is unclear if relative proportion of different Abs subclasses vary depending on the population. Both aspects need to be better explored to fully understand the humoral immune response induced in different populations. Indeed, differential functional activity based on Ig scaffold has been reported in literature for a variety of viral pathogens. For HIV, IgG3 enhances not only phagocytosis activity and binding to FcRIIIA compared to IgG1, but also the neutralization activity (8). Dimeric IgA (dIgA) was reported to have a higher neutralizing activity against SARS-CoV-2 compared with its monomeric counterpart (9). Whether different Ig scaffolds can influence Rotavirus-specific monoclonal antibodies (mAbs) remains to be determined.

Several human mAbs (HumAbs) directed at various rotavirus proteins were isolated and characterized from IgA-positive ascites, expressed in IgG1 scaffold, by Nair et al (5). Here, we expressed four of these antibodies (three targeting VP8* and one targeting VP7) in different antibody scaffolds and investigated the impact of antibody classes and subclasses on Rotavirus neutralization activity.

## Materials and methods

### mAbs selection

Four Rotavirus-specific antibodies targeting different Rotavirus epitopes and with different reactivity available form the literature (5) were selected for this work; three of these antibodies target the VP8* domain of VP4 (namely mAbs 8, 9, and 47), each exhibiting varying levels of neutralizing activity in the IgG1 scaffold, and one targets VP7 (mAb 46), known to neutralize effectively in the IgG1 scaffold (5).

### Plasmid DNA amplification, extraction, and quantification

Synthetic plasmids encoding for the antibody sequence of interest were purchased from TWIST Biosciences and GenScript. VH and VL regions of previously isolated mAbs (5) were inserted into a mammalian expression plasmid containing different constant regions of IgG1, IgG2, IgG3, IgG4, IgA1, IgA2 (**Supplementary Table 1**). Plasmids were amplified using *Escherichia coli (E. coli)* TOP10 competent cells (Sigma Aldrich) and plasmid DNA was retrieved using PureLink HQ Mini plasmid purification kit (Thermo Scientific™) following manufacturer’s instructions. Amplified DNA plasmids were quantified with Nanodrop2000 (Thermo Scientific™).

### Monoclonal antibodies expression in Expi293 mammalian cells

Mammalian cells (Expi293) were transfected with ExpiFectamine™ 293 Transfection Kit (Thermo Scientific™) following manufacturer’s instruction. Expression was achieved by co-transfection of plasmids containing heavy, light and, for dimeric IgA only, J-chain in mammalian cells. Briefly, each plasmid was diluted with 1.5 mL of Opti-MEM™ I Reduced Serum Medium, meanwhile 80 µL of ExpiFectamine™ 293 reagent was diluted with 1.4 mL Opti-MEM™ I Reduced Serum Medium and incubated for 5 minutes at RT, then the mix was added to the diluted plasmid and incubated 15–20 min at RT. Afterwards, the mixture was added to 25 mL Expi293 Expression medium containing 3x10^5^ cells/mL in a 125 ml flask and incubated overnight at 37°C, 8% CO_2_ with shaking at 125 rpm. From 18 to 22 hours post-transfection, 150 µL of ExpiFectamine™ 293 Transfection Enhancer 1 and 1.5 mL of ExpiFectamine™ 293 Transfection Enhancer 2 were added to each flask. Five to seven days after transfection, monoclonal antibodies were collected, centrifuging culture at 300 x g for 10 minutes at 4°C, filtering supernatant with 0.22 µm membranes (Millipore^®^ Stericap™, Gibco) and stored at 4°C for further analysis.

### Monoclonal antibodies purification

Antibodies were purified with ÄKTA avant (Cytiva). IgG1, IgG2 and IgG3 subclasses mAbs from supernatant were purified with a Hi-Trap Protein G column (1 and 5 mL, Cytiva), which contains a type III Fc receptor of Group G *streptococci* that binds to the Fc region of IgG subclasses. After the elution step in a 96-well format plate, all mAb containing wells were pooled together and buffer was exchanged to 1xPBS with PD-10 desalting columns (Cytiva).

IgA subclasses were purified with a Superose™ 6 column (Cytiva) by size exclusion chromatography (SEC). The selected column has high resolution capability for effective separation of molecules in the 10–300 kDa range. Following SEC, mAbs-containing fractions were pooled and further centrifuged to eliminate impurities at lower molecular weight with Amicon Ultra Centrifugal Filters (Merck Millipore). Filters utilized were of 30 kDa for monomeric mAbs and 100 kDa for dimeric ones. All mAbs were filtered through 0.2 µm membranes, aliquoted and stored at -80°C for further analysis.

### Quantification of monoclonal antibodies

Quantification of mAbs was performed using Nanodrop2000 spectrophotometer (Thermo Scientific™) with the laser at 280 nm of wavelength, accounting for the extinction coefficient of the different mAbs analyzed. Measurements were performed in triplicate, allowing for calculation of standard deviation (SD) and coefficient of variance (CV%). When CV% was lower than 30%, quantification was considered acceptable.

### Biolayer interferometry

VP8*-specific mAb IgG and IgA scaffolds binding to the PATH vaccine candidate P2-VP8*P[8] were analyzed using a Biolayer Interferometry (BLI) Technique (Octet^®^ R8).

For the analysis, mAbs were prepared at a concentration of 2 µg/mL, whereas P2-VP8*P[8] antigen was used at 10 µg/mL. The following layout was used in a 96-well BLI plate. Assay was performed by manufacturer’s instructions, using His1k biosensor to load the His-tagged VP8* protein.

### Rotavirus strains and cell lines

Rhesus monkey kidney cells (MA-104, ATCC) were propagated in complete medium (DMEM + 10% FBS + 1% Pen/Strep, Invitrogen) in a T75 flask and incubated at 37°C, 5% CO_2_ and split 1:12 v:v every 2–3 days.

Three different Rotavirus strains (Wa G1P[8], DS-1 G2P[4] and WI-61 G9P[8]) were purchased from American Type Culture Collection (ATCC) and propagated in MA-104 at low multiplicity of infection (MOI). Vials of purchased rotavirus stocks were thawed and activated with trypsin at a final concentration of 10 µg/mL for 1h at 37°C. Complete DMEM medium was removed from monolayer of MA104-cells culture (T75 flasks), and activated virus was diluted in DMEM without FBS, added to the flask and incubated for 1h at 37°C.

Following virus adsorption, inoculum was removed and DMEM media without FBS and trypsin was added. Cell culture was maintained for a week until a cytopathic effect was visible. Lysates from cell culture were frozen at -80°C and thawed several times to promote the release of Rotavirus particles into the supernatant. Debris were then removed by centrifugation (300 x g for 3 minutes) and RV clarified stocks were titered, aliquoted, and stored at -80°C (10).

### Neutralization assay

On day 0, MA-104 cells (3x10^3)^ were plated in each well of 384-well plates, in complete medium and incubated at 37°C 5% CO_2_ for 72 hours. Recombinant mAb dilutions were prepared with a starting concentration of 20 µg/mL (10 µg/mL, final dilution) and were serially diluted (1:3 for 15 points) with DMEM media without FBS in a 384-well plate.

On day 3, a stock of viruses was activated with 10 µg/mL of trypsin for 1 hour at 37°. Following activation, virus dilutions were prepared in DMEM media without FBS: Wa G1P[8], Wi-61 G9P[4] DS-1 G2P[8] at 1:20, 1:40 and 1:100, respectively. Next, 25 µL of mAb was incubated with 25 µL of virus in each well for 1 hour at 37°C. To infect MA-104 monolayer cells, the mAb-RV mixture was added in a 384-well plate and incubated at 37°C/5% CO_2_. Sixteen hours after infection, supernatant was removed and cells were fixed and permeabilized with 25 µL of Cytofix/Cytoperm™ Fixation Kit (BD Biosciences) at room temperature for 20 min. Cells were washed with 80 µL of Perm Wash (1x, BD Biosciences) and 25 µL of polyclonal goat anti-Rotavirus antibodies (Millipore) 1:250 in PBS/0.1% Tween 20/0.1% BSA was added to each well of the plates for 1 hour.

After a wash, 25 µL of rabbit anti-goat IgG antibody F(ab’)2, FITC Conjugate (Sigma Aldrich) diluted 1:500 in PBS/0,1% Tween20/0.1% BSA were added to the wells in each plate for 1 hour in the dark. Following incubation, the secondary antibody was removed and stained cells were washed twice with 80 µL of PBS/0.1% Tween20, and then with 80 µL of PBS. Images of the stained cells were acquired with a live-cell imaging Tecan’s Spark^®^ reader. A 4-parameter nonlinear regression analysis was applied to raw data (number of infected objects versus Log mAbs concentration) and IC_50_ (mAb concentration to neutralize 50% of infection) was measured using GraphPad Prism (v. 10.2.3).

### Statistical analysis

Neutralization results were obtained from three independent experiments, each one performed in triplicate, using serially diluted mAbs. For statistical analysis, two times the concentration of the highest concentration tested (10 µg/mL) was assigned to non-neutralizing mAbs. To determine differences of mAb scaffolds neutralizing activity compared to the IgG1 scaffold a Kruskal-Wallis Test was performed for each mAb and strain tested. Differences were considered significant when p-value was inferior to 0.05. Moreover, to determine the impact of mAb scaffold on neutralizing Rotavirus multiple comparison analysis was performed as well. All analysis were performed with GraphPad Prism (v. 10.2.3).

## Results

### Binding of VP8*-specific mAbs do not differ in different scaffolds

**Figure 1:**
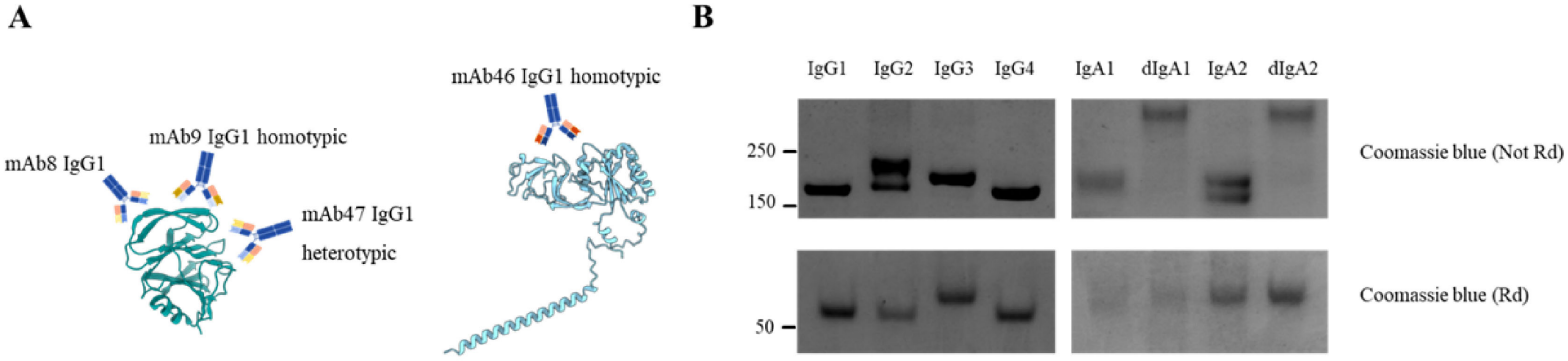
mAbs binding information and purification results. **(A)** represent the specificity of selected mAbs, while in **(B)** are reported the Coomassie blue stain of purificated mAb9 versions obtained. In the top 2 images the whole mAbs are reported, while in the bottom 2 images the heavy chains of each mAb versions are shown. Molecular weight in kDa is reported on the left side of images. mAb9 was selected as representative of obtained mAbs. Not Rd stands for non-reducing condition, while Rd stands for reducing condition in which SDS-Page was run.

The four selected antibodies target VP7 (mab46) and VP8 (mAbs 8, 9, and 47) in three distinct epitopes as it is suggested by their different neutralization activity and reactivity on different Rotavirus strains as well (**Supplementary Table 2**) (5). All mAbs were produced in IgG1, IgG2, IgG3, IgG4, IgA1, and IgA2 scaffolds, with IgAs in monomeric and dimeric forms. Purity of Abs was determined by SDS-Page, followed by Coomassie blue staining. Analysis showed only the bands at the expected molecular weight (about 160 kDa for monomeric mAb and 300 kDa for dimeric ones in non-reducing condition, **Figure 1**). Except for mAb46 that could not be expressed in the IgA1 and dIgA1 scaffolds, the remaining mAbs were successfully expressed in the eight scaffold versions, resulting in a collection of 30 mAbs.

The binding affinity of all IgG and IgA isoforms of VP8*-specific mAbs by biolayer interferometry (BLI, Octet) is presented in **Supplementary Table 3**. All versions of mAb9 had a K_dis_ around 10^-7^, while mAb8 IgG2 and IgA2 have a K_dis_ around 10^-3^, as well as mAb47 IgG1, IgG2 and IgA2. Intriguingly, dIgA2 of both mAbs possess better K_dis_ of 4.422*10^-4^, and 3.553*10^-7^, for mAb47 and 8, respectively. Due to failure of the expression of the recombinant VP7, binding of VP7-targeted mAbs was not assessed.

### mAbs with lack of neutralizing activity in IgG1 acquire it in other scaffolds

**Figure 2:**
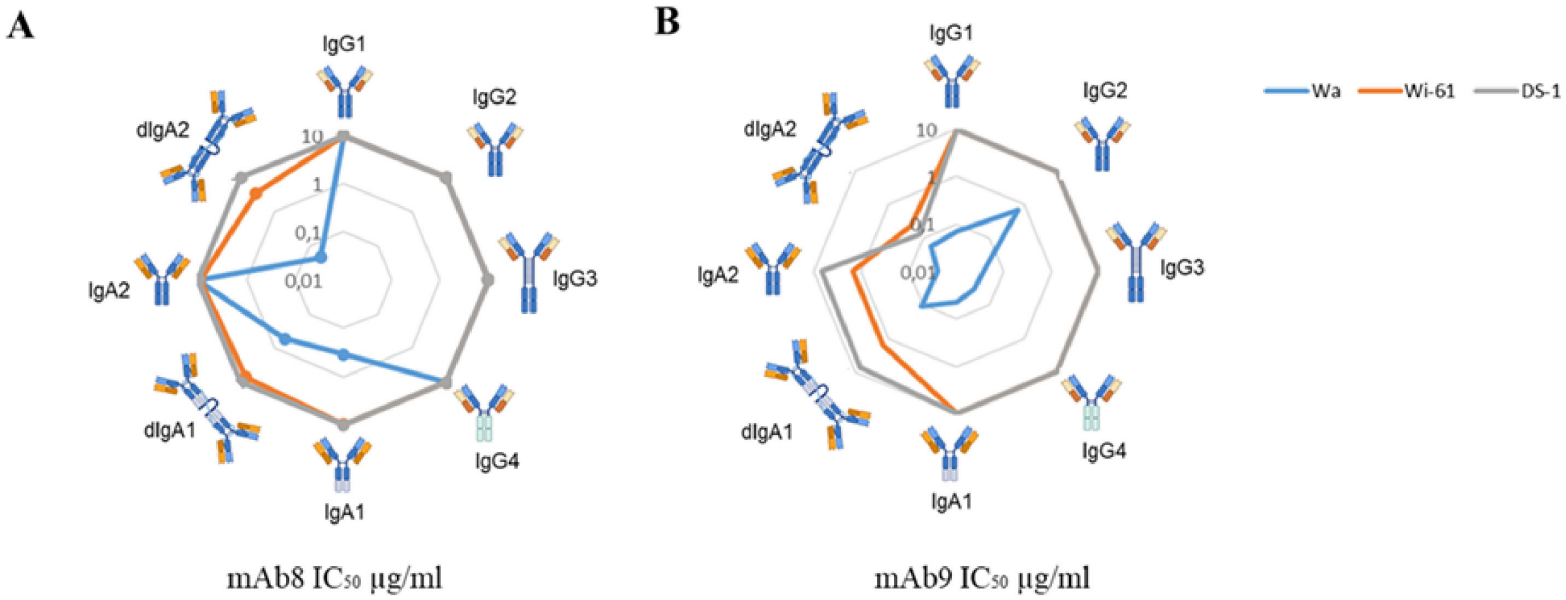
IC_50_ of VP8*-specific mAb8 and mAb9 expressed in different scaffolds on three different Rotavirus strains. In the graphs are reported the IC_50_ (µg/ml) of neutralization activity of each isotype of IgG and IgA of mAb8 **(A)**, and mAb9 **(B)** against Wa (light blue), Wi-61 (orange) and DS-1 (gray) strains.

VP8*-specific mAb8, previously reported as non-neutralizing in IgG1 scaffold (5) at the tested concentration, did not prevent infection in our *in vitro* model either when expressed in the other IgG scaffolds or in IgA2 monomeric form. However, mAb8 demonstrated strain-dependent neutralization against Wa G1P[8] when expressed in IgA1, dIgA1 and dIgA2 scaffolds (**Figure 2A**).

The increased neutralization activity observed for mAb8 in both monomeric and dimeric forms of IgA compared to IgG subclasses was also observed with mAb9. We noted significant neutralizing activity with mAb9 dIgA2 compared to IgG1 (p-value = 0.01), with dIgA1 compared to IgG1 against the Wi-61 G9P[8] strain (p-value = 0.03), dIgA2 compared to IgG1 (p-value = 0.003), dIgA2 against the DS-1 G2P[4] strain (p-value = 0.02) (**Figure 2B**). Moreover, mAb9 expressed in different scaffolds were always able to neutralize Wa strain, with lower neutralizing ability induced by IgG2 mAbs (p-value = 0.04).

### Neutralizing mAbs are less impacted by different Ig scaffold

**Figure 3:**
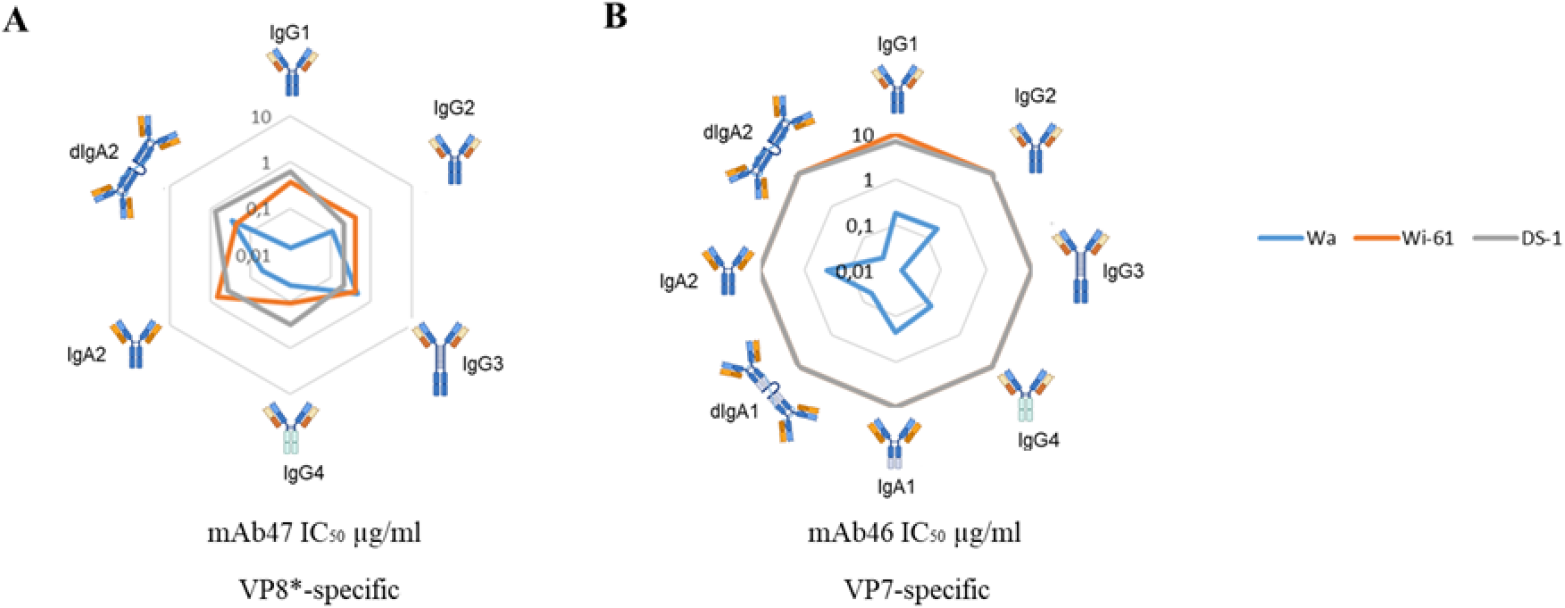
IC_50_ of mAb 47 and 46 expressed in different scaffolds on three different Rotavirus strains. In the graphs are reported the IC_50_ (µg/ml) of neutralization activity of each isotype of IgG and IgA of mAb47 **(A)**, and mAb46 **(B)** against Wa (light blue), Wi-61 (orange) and DS-1 (gray) strains.

VP8*-specific mAb47 was able to effectively neutralize all the Rotavirus strains in the different scaffolds tested. No substantial differences in neutralizing activity have been observed between the different scaffolds across all tested strains. However, for the Wa strain it was possible to appreciate stronger neutralizing activity of mAb47 in IgG1 (IC_50_ = 0.014 µg/ml), IgG4 (IC_50_ = 0.05 µg/ml), and IgA2 (IC_50_ = 0.05 µg/ml) forms. Interestingly a generally enhanced activity of dimeric IgA (IC_50_ = 0.023 µg/ml) compared to monomeric IgA (IC_50_ = 0.068 µg/ml) forms against the Wi-61 G9P[8] strain was observed, with a trend opposite to what was observed for the same mAbs against Wa and DS-1 strains (**Figure 3A**).

The neutralizing activity of VP7-specific mAb46 was initially characterized as G1-type-specific, reacting to Wa G1P[8] serotype (5). Consistent with earlier findings, our study confirmed that mAb46 in IgG1 scaffold neutralized effectively only the Wa G1P[8] Rotavirus strain; however, we observed a weak (IC_50_ = 7.032 µg/ml) previously undescribed neutralization activity against the DS-1 G2P[4] Rotavirus strain (**Figure 3B**). mAb46 expressed in the various scaffolds was able to effectively neutralize infection by the Wa strain, with a more pronounced – although not statistically significant (p-value > 0.05) – activity in IgG3 (IC_50_ = 0.013 µg/ml), dimeric IgA1 (IC_50_ = 0.054 µg/ml) and IgA2 (IC_50_ = 0.024 µg/ml) forms. In contrast, even when expressed in the various scaffolds, mAb46 was unable to neutralize infection by DS-1 and Wi-61 strains.

## Discussion

For influenza, RSV, HIV, SARS-CoV-2 and related viruses, the neutralization of infection is not solely dependent on the Fab portion. Indeed, factors such as valency, hinge flexibility, Fc glycosylation, and the orientation of Fc/Fab domains influence the binding to viral target epitopes and the blocking of viral infection (8, 11–16).

Specifically, IgG3 antibodies targeting the receptor binding domain (RBD) have been shown to increase the potency of antibodies compared to IgG1, IgG2, or IgG4 formats (17). Additionally, IgG3 antibodies targeting the RBD of SARS-CoV-2, or the hemagglutinin of influenza virus, demonstrate more efficient binding and neutralization of antigenically drifted strains than IgG1 or IgG2 antibodies (11). However, in our study, IgG3 did not emerge as the optimal scaffold for Rotavirus neutralization. Notably, the VP7-specific mAb46, previously reported to neutralize Wa G1P[8], did not exhibit significantly improved neutralization in IgG3 scaffolds compared to IgG1 when tested against the same strain. However, its activity was comparable to that of the dimeric IgA forms, which proved to be the most potent in our tested panel (**Figure 3**). Indeed, the dIgA1 and dIgA2 scaffolded versions exhibited greater neutralizing activity than their monomeric IgA1, IgA2 or IgG1 counterparts (**Figure 2**). This observation aligns with findings by Marcotte and colleagues, who reported that IgG1, monomeric IgA1, and dimeric IgA1 bind the SARS-CoV-2 spike protein with different geometries, resulting in neutralization of drifted variants primarily mediated by dimeric and secretory IgA variants (18). Furthermore, Wang and colleagues reported that dIgA targeting the SARS-CoV2 receptor binding domain offers superior neutralization compared to IgG1 scaffold (9). Moreover, influenza viruses are also more susceptible to dimeric IgA due to their enhanced avidity and neutralizing capabilities resulting from multimerization (9, 19).

Additionally, we observed that the impact of the Ig scaffold was more pronounced for Rotavirus VP8*-specific mAbs exhibiting weak or absent neutralization. Conversely, mAbs previously reported as neutralizing were minimally impacted by scaffold exchange. The neutralization activity of VP8*-specific mAb47, previously reported as neutralizing against Wa G1P[8] (5), did not vary significantly amongst its mAb versions across all the three tested strains. Conversely, mAb8 and mAb9 benefitted from the IgA variants, as the only mAb8 versions mediating neutralization on Wa G1P[8] were IgA, and neutralization was also mediated by mAb9 IgA variants on Wi-61 and DS-1 strains (**Figure 2**). These findings may be attributed to differences in mAbs avidity, which impacts their neutralization capacity, consistent with observations in other viruses (20–22) (23) (24). Therefore, the higher avidity of dIgA structures of mAb8 and mAb9 could enable the recognition of multiple VP8* epitopes on the same viral particle, thereby mediating neutralization only in these mAbs scaffolds.

We noted that mAb8 forms capable of neutralizing the Wa strain (dimeric IgA1, IgA2, and monomeric IgA1) possess a K_dis_ in the order of 10^-7^ 1/s (**Supplementary Table 3**). Nonetheless, the IgG1 and IgG3 forms of this mAb showed a similar K_dis_ but failed to neutralize Rotavirus infection. Indeed, considering solely the K_dis_ is insufficient to explain the differential neutralization activity observed in this study. Dimeric IgA versions possess different avidity than their monomeric counterparts. As extensively reported in literature concerning other viruses (18, 25, 26), without modeling avidity, standard K_dis_ measurements on multivalent surfaces cannot be equated with intrinsic neutralization activity (27).

Although viral protein epitope mapping and detailed kinetic binding analysis of each mAb to its target were beyond the scope of this study, future research will focus on these aspects. Indeed, a comprehensive understanding of the binding kinetics of mAbs in various scaffolds could shed further light on their ability to neutralize diverse Rotavirus serotypes. In our study non-entry neutralization, which is known to play a role in Rotavirus infection (28), was not investigated, as we specifically focused on the ability of expressed mAbs to inhibit viral entry. The Fc portion of mAbs can trigger various activities of innate immune system, depending on the specific Fc-receptors through which they are recognized (29, 30). Future research will also focus on investigating this additional neutralization mechanism and Fc-mediated effector functions of immune cells.

Since type-specific and cross-reactive antibodies do not always correlate with protection (31), studies on protection mechanism are crucial for identifying the optimal antibody isoform to predict vaccine effectiveness. Determining the mechanisms of Rotavirus neutralization by IgG and IgA subclasses in more complex *in vitro* and *in vivo* models may more closely replicate protection mechanisms observed in humans. Indeed, these different mAb versions can be applied in primary cultures seeded in transwells to assess their neutralization ability when present in the apical or basolateral chamber. Such experiments, conducted in a more physiologically relevant *in vitro* model, will help unravel the effect of serum IgGs and IgAs on the inhibition of Rotavirus entry into the gut lumen. Furthermore, depending on the vaccine platform (32) or adjuvant used (33, 34), varying quantities and qualities of antibodies are elicited. Therefore, a comprehensive understanding of the role each antibody subclass plays in inducing neutralizing activity can guide improved vaccine design to target the response against the desired subclass(es). Eventually, the generated mAb versions could serve as valuable tools to establish standards for human serological assays requiring Rotavirus-specific mAbs.

In conclusion, by expressing a panel of mAbs targeting diverse Rotavirus epitopes, we have demonstrated that mAbs directed against the same epitopes can exhibit varying neutralizing activity when expressed in different scaffolds, and their effectiveness can differ across Rotavirus strains. It is important to note that the observed patterns were specific to each mAb’s targeted epitope, implying that this behavior is epitope-dependent and cannot be universally applied to all Rotavirus-specific antibodies. The overall increased neutralizing activity of IgA variants compared to IgG counterparts was statistically significant only for mAbs exhibiting poor neutralization activity in IgG1 format targeting VP8*. To generalize these findings, larger and more comprehensive studies are warranted. Nonetheless, for the first time in the Rotavirus field, we provide proof-of-concept that the same antibody, when expressed in different subclasses, displays distinct functionality.

## Data Availability

The original contributions presented in the study are included in the article/**Supplementary Material**. Further inquiries can be directed to the corresponding author.
