# Supplementary material for: Different Fc scaffolds enhance the breadth of in vitro neutralization of the same Fab against different Rotavirus strains
Source: Front Immunol. 2026 Jan 9;16:1709107. doi: 10.3389/fimmu.2025.1709107 (PMC12829111; doi:10.3389/fimmu.2025.1709107)
Supplement: Supplementary file 2 [file Table2.docx]

**Different Fc scaffolds enhance the breadth of *in vitro* neutralization of the same Fab against different Rotavirus strains**

M.R. Miranda Echague^1,2+^, G. Vezzani^1*+^, E. Morandi^1^, M. Della Peruta^1^, M. Scordio^1^, T.A.C. Reyes^1^, D. Oldrini^1^, M. Iturriza-Gómara^1^, R.J. Loomis^1^, O. Rossi^1^

^1^ GSK Vaccines Institute for Global Health (GVGH), Siena, Italy

^2^ Fondazione Biotecnopolo di Siena, Siena, Italy

+ Authors contributed equally to the work

***Correspondence:** Giacomo Vezzani, [giacomo.x.vezzani@gsk.com](mailto:giacomo.x.vezzani@gsk.com)

**Keywords:** Rotavirus, immunology, vaccines, neutralization assay, mAbs scaffold


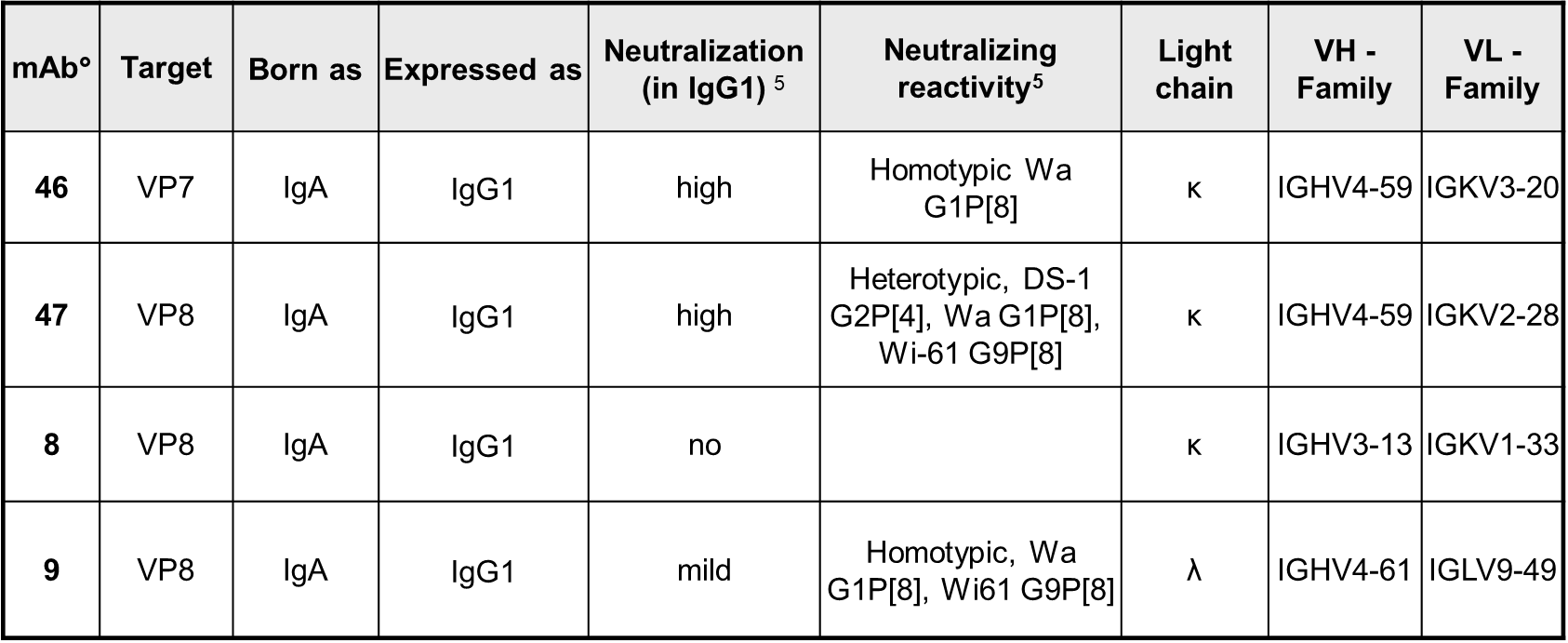


**Supplementary Table 2. List of monoclonal antibodies utilized in this work.** In each row are reported selected mAbs ID. In each column are reported, from left to right: mAbs, target protein, isolated and expressed subclass, neutralization potency, neutralizing reactivity, light chain, and gene family of variable regions composing the mAbs.
