# Supplementary material for: Different Fc scaffolds enhance the breadth of in vitro neutralization of the same Fab against different Rotavirus strains
Source: Front Immunol. 2026 Jan 9;16:1709107. doi: 10.3389/fimmu.2025.1709107 (PMC12829111; doi:10.3389/fimmu.2025.1709107)
Supplement: Supplementary file 3 [file Table3.docx]

**Different Fc scaffolds enhance the breadth of *in vitro* neutralization of the same Fab against different Rotavirus strains**

M.R. Miranda Echague^1,2+^, G. Vezzani^1*+^, E. Morandi^1^, M. Della Peruta^1^, M. Scordio^1^, T.A.C. Reyes^1^, D. Oldrini^1^, M. Iturriza-Gómara^1^, R.J. Loomis^1^, O. Rossi^1^

^1^ GSK Vaccines Institute for Global Health (GVGH), Siena, Italy

^2^ Fondazione Biotecnopolo di Siena, Siena, Italy

+ Authors contributed equally to the work

***Correspondence:** Giacomo Vezzani, [giacomo.x.vezzani@gsk.com](mailto:giacomo.x.vezzani@gsk.com)

**Keywords:** Rotavirus, immunology, vaccines, neutralization assay, mAbs scaffold

| **Sample ID** | **Response** | **K_dis_ (1/s)** |
| --- | --- | --- |
| mAb47 IgG1 | 0.0969 | 1.000E-03 |
| mAb47 IgG2 | 0.0344 | 5.496E-03 |
| mAb47 IgG3 | 0.1251 | 3.539E-07 |
| mAb47 IgG4 | 0.1254 | 2.693E-07 |
| mAb47 IgA2 | 0.1251 | 1.000E-03 |
| mAb47 dIgA2 | 0.4040 | 4.422E-04 |
| mAb8 IgG1 | 0.7306 | 4.875E-07 |
| mAb8 IgG2 | 0.1449 | 1.000E-03 |
| mAb8 IgG3 | 0.3561 | 2.864E-07 |
| mAb8 IgG4 | 0.4645 | 6.977E-05 |
| mAb8 IgA1 | 0.1931 | 3.781E-07 |
| mAb8 dIgA1 | 0.0110 | 2.441E-07 |
| mAb8 IgA2 | 0.1285 | 1.000E-03 |
| mAb8 dIgA2 | 0.3852 | 3.553E-07 |
| mAb9 IgG1 | 0.4531 | 4.418E-07 |
| mAb9 IgG2 | 0.2689 | 2.840E-07 |
| mAb9 IgG3 | 0.3535 | 4.104E-07 |
| mAb9 IgG4 | 0.4066 | 2.674E-07 |
| mAb9 IgA1 | 0.4906 | 4.000E-07 |
| mAb9 dIgA1 | 0.4289 | 2.999E-07 |
| mAb9 IgA2 | 0.7809 | 3.979E-07 |
| mAb9 dIgA2 | 0.5433 | 4.752E-07 |
| mAb46 IgG1 | N/A | N/A |
| mAb46 IgG2 | N/A | N/A |
| mAb46 IgG3 | N/A | N/A |
| mAb46IgG4 | N/A | N/A |
| mAb46 IgA1 | N/A | N/A |
| mAb46 dIgA1 | N/A | N/A |
| mAb46 IgA2 | N/A | N/A |
| mAb46 dIgA2 | N/A | N/A |

**Supplementary Table 3. Binding of VP8*-specific mAbs.** From left to right: sample ID, Antigen loading/mAb binding ratio response, and dissociation constant (K_dis_), of purified mAb obtained in PBS**.**
